# High-Risk HPV Oncoproteins and PD-1/PD-L1 Interplay in Human Cervical Cancer: Recent Evidence and Future Directions

**DOI:** 10.3389/fonc.2020.00914

**Published:** 2020-06-30

**Authors:** Soumaya Allouch, Ahmed Malki, Asma Allouch, Ishita Gupta, Semir Vranic, Ala-Eddin Al Moustafa

**Affiliations:** ^1^College of Medicine, QU Health, Qatar University, Doha, Qatar; ^2^Biomedical Science Department, College of Health Sciences, QU-Health, Qatar University, Doha, Qatar; ^3^Biomedical Research Center, Qatar University, Doha, Qatar

**Keywords:** high-risk HPV, oncoproteins, PD-1/PD-L1, cervical cancer, immunotherapy

## Abstract

Cervical cancer is the fourth most common malignancy in women worldwide and a leading cause of cancer-related mortality in developing countries. Important etiological factors in this cancer are high-risk human papillomaviruses (HPV), as roughly 96% of cervical cancer cases are positive for these oncoviruses. On the other hand, it has been recently pointed out that E6/E7 oncoproteins of high-risk HPV can upregulate the programmed cell death-1/programmed cell death-ligand 1 (PD-1/PD-L1) axis. Likewise, several recent reports showed that checkpoint blockades targeting PD-1/PD-L1 pathways have achieved efficient clinical responses via suppressing cancer progression and improving survival in several types of human cancers including metastatic cervical cancer. In this review, we summarize recent advances in our understanding of the PD-1/PD-L1 signaling pathway and its interaction with high-risk HPV and their oncoproteins, which could have an important impact on the management of HPV-associated cancers including cervical.

## Introduction

The World Health Organization (WHO) categorizes cervical cancer as the fourth most common cancer in females, accounting for 30% of cancer-mortality cases (1). In contrast, in developing countries, cervical cancer is classified as the most frequent gynecological cancer (2). The noted increase in disease prevalence in developing countries is partially attributed to immune-compromising conditions including HIV, which in turn is associated with a higher risk of persistent and multiple human papillomavirus (HPV) infections (3, 4). HPV is a non-enveloped double-stranded DNA virus that encompasses more than 150 types. On the basis of oncogenicity, HPVs are classified into high-risk (16, 18, 31, 33, 35, 39, 45, 51, 52, 56, 58, 59, 68, 69, 82) and low-risk (6, 8, 11, 40, 42, 43, 44, 54, 61, 72) (5), of which high-risk HPV types 16 and 18 are the most widespread carcinogenic strains to humans. Additionally, results from case–control studies report a convincing association between high-risk HPV types 31, 33, 35, 39, 45, 51, 52, 56, 58, 59, and 66 and carcinogenesis (5). To date, HPV, is the most common sexually transmitted viral infection worldwide; additionally, it is considered as the main causative agent of cervical intraepithelial neoplasia (CIN) and cervical cancer (6, 7).

The HPV DNA genome encodes for eight proteins, of which E5 and E6/E7 of high-risk HPV are reported oncogenes possessing transforming characteristics (8), while E5 oncoprotein affects cell alteration leading to carcinogenesis (9–11). More specifically, E5 oncoprotein functions by enhancing the expression of epidermal growth factor receptor-1 (EGFR-1) (12) and increases phosphorylation of protein kinase B (AKT), thereby activating the MEK/ERK1/2 pathway, thus inducing HPV-associated carcinogenesis (13, 14). Concomitantly, Yan et al. reported that in cervical cancer cells, inhibition of the PI3K/Akt pathway reduces epithelial–mesenchymal transition (EMT) as well as cellular invasion and migration, providing a potentially valuable therapeutic target for cervical cancer (15). On the other hand, gene transfer studies showed that in HPV16- and HPV18-induced cervical carcinoma, E6 and E7 are the chief viral oncoproteins responsible for cell cycle deregulation (16). The effects of E6 protein are mainly mediated through two pathways. Firstly, E6 serves as a ligand to p53, enhancing degradation via ubiquitin (17, 18). In healthy individuals or in HPV-negative cells, degradation of p53 is mediated through the ubiquitin–proteasome system (19). Secondly, E6 triggers telomerase activity contributing to the uncontrolled proliferative capacity of cancerous cells (20). Equally, E7 oncoprotein exerts its action by binding to several members of the retinoblastoma (Rb) family of tumor suppressor proteins, mainly hypo-phosphorylated form of pRb, resulting in destabilization of the Rb/E2F complex, which in turn leads to an increase in gene transcription and initiate cell proliferation (21).

On the other hand, immune checkpoints are essential mechanisms that function under physiological conditions averting autoimmunity and minimizing allergic reactions (22). Programmed cell death-1 (PD-1) is a type I transmembrane protein receptor that upon interacting with its ligands, PD-L1, and PD-L2, triggers a cascade of downstream signals and suppresses T cell activation (23). Since CD8+ T cells play a vital role in eliminating cancer cells, their inhibition interferes with cancer eradication and permits its immune escape (24). In cervical cancer, multiple studies have emphasized on the independent role of the PD-/PD-L1 pathway in cancerous cell proliferation and growth (25, 26) and the involvement of E5 and E6/E7 oncoproteins in the pathogenesis of cervical cancers (27, 28). However, the interrelation between high-risk HPV oncoproteins (E5, E6/E7) and PD-1/PD-L1 pathways and their potential synergic effect are yet to be fully elucidated. Furthermore, identification of potential therapeutic options designed to target HPV-PD-1/PD-L1 interaction in cervical cancers and CIN is essential given the prominence of HPV infections in this type of cancers. This review aims to underline the interrelation between high-risk HPV oncoproteins and PD-1/PD-L1 in the pathogenesis of cervical cancer, which could reinforce the role of PD-1/PD-L1 inhibitors as one of the main therapies for the management of HPV-positive human carcinomas including cervical.

## PD-1 and PD-L1 Pathway

As we mentioned above, the role of high-risk HPV in cervical carcinogenesis is well-established and documented (29, 30). On the other hand, the emergence of immunotherapy coupled with the exponential increase in understanding therapy-induced host immune response in hindering tumor proliferation played a pivotal role in the identification of novel immune checkpoint inhibitors for treatment of several types of cancers (25, 31, 32). In physiological conditions, immune checkpoints are thought to be essential in regulating the immune system, thereby preventing the development of autoimmune diseases (33). In this context, the PD-1 receptor is thought to be an immune checkpoint mediator, subduing tumor-induced immunity (34).

In healthy individuals, the PD-1 pathway regulates antigen-mediated inflammatory response to ensure minimal damage to healthy tissues (35). Notably, in the presence of a recognizable antigen expressed by the major histocompatibility complex (MHC), an inflammatory response is triggered through the activation of T cells and recruitment of cytokine-producing cells (34). Immune tolerance is elicited following cytokine-induced PD-L1 expression in tissues and the activation of PD-1 protein on T-cells (34). Once activated, the PD-1/PD-L1 pathway inhibits T-cell receptor (TCR) signal transduction and CD28–CD8 co-stimulation, thus hindering T cell activity (36). On the other side of the spectrum, toll-like receptors (TLR) function through sensing common pathogen features and activating innate immunity. Drug-induced activation of TLR9 was proven to moderately improve immunogenic tumor sensitivity and augment innate immunity to promote tumor regression, thus indicating plausible potential synergetic effects between TLR9 agonist and PD-1 inhibitors (37).

## PD-1 Checkpoints and Cervical Cancer

Several studies showed an elevation of PD-L1 in a variety of cancers, including cervical. Immunohistochemistry studies were used to determine PD-L1 expression, and the staining intensity was used to quantify the levels of PD-L1 in tumor sections (38, 39). Elevated PD-L1 expression inhibits T cell activity and thereby favors a state of immune resistance (40). Further, a direct correlation between PD-L1 overexpression and poor overall survival in patients with cervical cancer was reported (41). Markedly, Wang et al. reported an overexpression of PD-L1 levels in CIN 1–2 lesions (20/21 = 95%) as opposed to histologically normal cervical epithelia (0/55) (42). On a similar note, Meng et al. and Feng et al., respectively, reported an overexpression of PD-L1 in more than 60% (59/97) and 45% (31/66) of patients with cervical cancer (39, 43). Additionally, PD-L1 expression was found in 34.4–96% of cervical carcinomas as opposed to histologically normal cervical tissues in which PD-L1 expression was minute (26, 44, 45). Similarly, Reddy et al. reported an increase in PD-L1 expression by 29% in adenosquamous carcinomas, 17% in endocervical carcinomas, and 38% in squamous cell carcinomas (SCCs) (46). Furthermore, Heeren et al. found a higher abundance of PD-L1-positive immune cells in close proximity to metastatic tumors as compared with paired primary tumors. The quantification was done based on the presence or absence of PD-L1-positive tumor-infiltrating cells as well as the accumulation of immune cells around tumor fields forming a PD-L1-positive cordon. Additionally, the authors reported PD-L1-positive tumor-associated macrophages, expressing CD163, and/or CD14 (47). This implies the important role that PD-1/PD-L1 axis plays in hindering immunity against cervical cancer. Remarkably, upregulated levels of PD-L1 in SCCs are associated with poorer disease-free and disease-specific survival rates in comparison to those with normal or minute PD-L1 levels (47). Moreover, presence of PD-L1-positive tumor-associated macrophages correlates with worst disease-specific survival rates in adenocarcinoma patients (47). Evidently, the PD-1/PD-L1 pathway plays an important role in the pathogenesis of cervical cancer.

The illustrated PD-1 mechanism of action and the role it plays in the pathogenesis of a variety of cancers coupled with its unique structure—resembling that of cytotoxic T-lymphocyte-associated antigen 4 (CTLA-4) but with a unique ligand specificity and biological functionality—makes it an exceptional drug target (48). Unlike CTLA-4 ligands, earlier studies pointed out that PD-1 ligands are selectively expressed in various cancers as well as within tumor microenvironments (49, 50); specifically, PD-1 was found to be quite prominent in various solid and hematological malignancies (49–54). An *in vitro* study by Fife et al. revealed that antibody-mediated inhibition of PD-L1 binding to PD-1 resulted in lower T cell motility and enhanced T cell-dendritic cell interaction (55). Together, these findings support the use of PD-1 inhibitors as a promising strategy for tumor immunotherapy.

Understanding the pathways through which PD-L1 checkpoint activation leads to the development and progression of solid tumors provides a path to investigate the effects of PD-L1 inhibitors on solid tumor regression. Phase 3 clinical trials revealed a statistically significant increase in overall survival in myeloma patients receiving nivolumab (PD-L1 inhibitor) with 73% overall survival as compared to 42% for those who received dacarbazine (standard treatment) (56). Administration of various doses of pembrolizumab in patients with recurrent metastatic cervical cancer showed an overall response rate (ORR) of 14.33–17% (57, 58). Similarly, in patients with recurrent or metastatic HPV-related cancers (19 cervical and five vaginal/vulvar carcinomas, CheckMate358 study, NCT02488759), administration of nivolumab showed an ORR of 26% in patients with cervical cancer (59). Notably, the response to nivolumab was unrelated to PD-L1 status or previous treatments. Thus, the use of PD-1 inhibitors for cervical cancer is a promising treatment strategy.

In this context, pembrolizumab, an immune checkpoint inhibitor, represents a full-length human IgG4/kappa monoclonal antibody that is directed against the PD-1 protein (60, 61) and has been approved by the FDA as a second-line treatment for recurrent or metastatic carcinomas of the cervix, non-small cell lung, and urothelial as well as malignant melanoma (60). Pembrolizumab (Keytruda) was approved for the treatment of patients with recurrent and/or metastatic cervical cancer in 2018 based on the KEYNOTE 158 (NCT02628067) Phase II study which involved 98 patients with recurrent and/or metastatic cervical carcinomas (62). The objective response rate (ORR) among 77 patients was achieved in 14.3% including 2.6% complete responses and 11.7% patients having partial responses (62). Of note, the FDA also concurrently approved the PD-L1 immunohistochemistry 22C3 pharmDx test (Dako Agilent) as a companion diagnostic test to guide the patient selection process for pembrolizumab treatment (63). This is critically important since pembrolizumab as a single agent exhibits a limited efficacy in recurrent and/or metastatic setting in an unselected patient population (61). Moreover, an ongoing phase III trial (KEYNOTE-826 phase III trial, NCT03635567) aims to treat advanced or recurrent cervical cancer in the first line using pembrolizumab or a placebo plus one of four platinum- and taxane-based chemotherapy regimens (61). Notably, patients are being stratified based on PD-L1 expression (combined positive score ≥1) by immunohistochemistry (62, 63).

Given that clinical benefits of pembrolizumab in cervical cancer are still sparse and limited, there is an unmet need for more trials and studies that explore the role of pembrolizumab in addition to other immune checkpoint inhibitors (e.g., PD-1 (nivolumab and cemiplimab) and PD-L1 inhibitors (e.g., durvalumab, avelumab, and atezolizumab) (64). A combinatorial approach with immune checkpoint inhibitors is also warranted (65). This is particularly important given that immune suppression (impaired cellular response) caused by the activation of the inhibitory axis PD-1/PD-L1 strongly favors persistent HPV infections, viral integrations into the cervical epithelium, and concomitant expression of the key viral oncoproteins such as E6 and E7 proteins (64). In addition, a combined treatment of immune checkpoint inhibitors with other therapeutic modalities (e.g., bevacizumab, conventional chemotherapy, radiotherapy) is also a huge challenge.

## HPV Oncoproteins and PD-1/PD-L1 Interaction in Cervical Cancer

**Figure 1:**
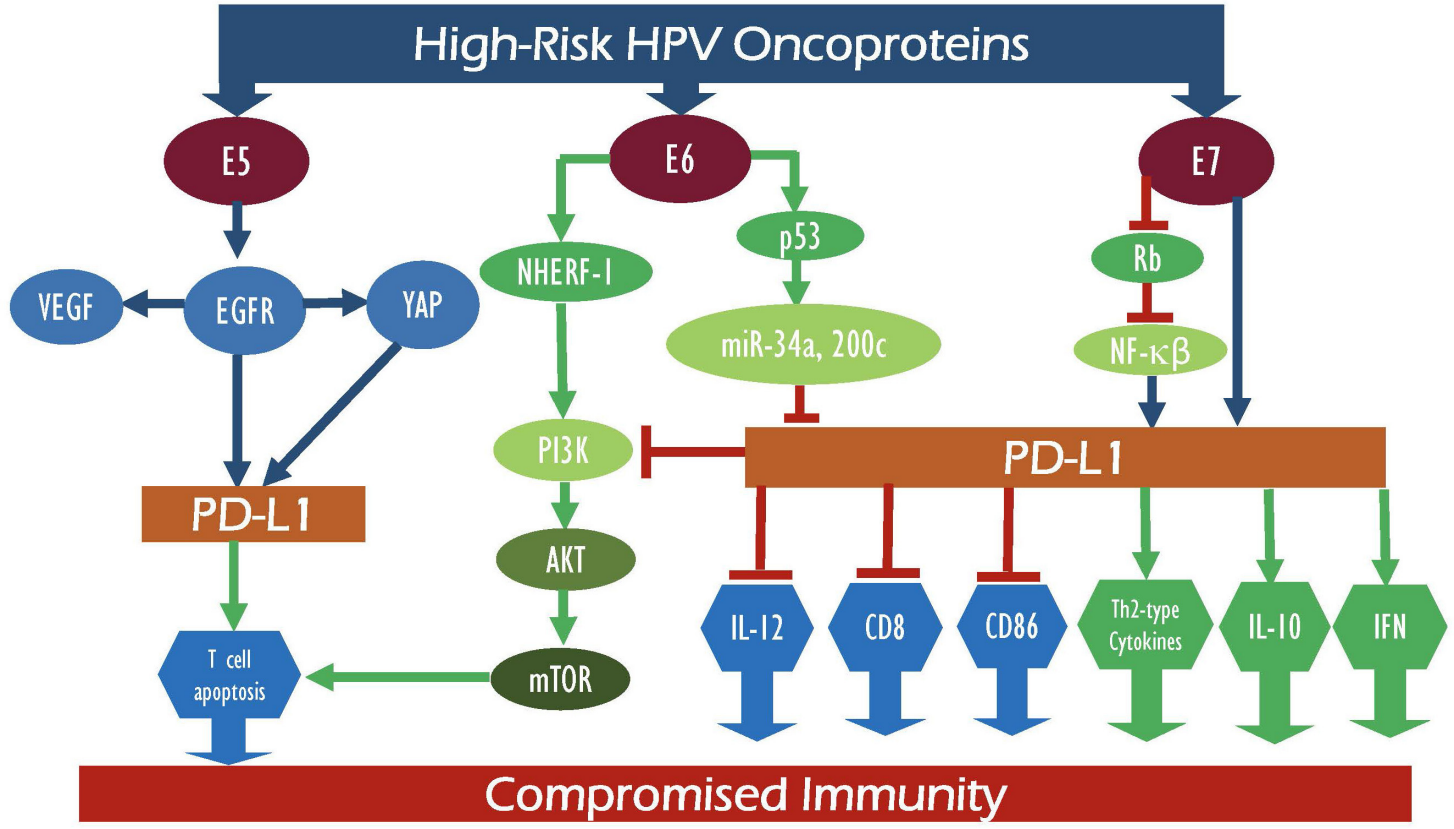
Schematic outline showing the potential interaction between E5, E6, and E7 of high-risk HPV oncoproteins with their intermediate proteins as well as their signaling pathways which can deregulate PD-1 and PD-L1 expression patterns, consequently leading to compromised immunity.

In the case of cervical cancer, high-risk HPVs are a determining factor in its pathogenesis; continual HPV infection is associated with pathogenesis of cervical cancer and is correlated with its prognosis. This, coupled with the significance of the PD-1/PD-L1 axis in cervical cancer etiology, has made it crucial to investigate the interrelation between E5 and E6/E7 oncoproteins and the PD-1/PD-L1 pathway in the pathogenesis of cervical cancer (Figure 1). Research has shown a significant association between HPV positivity and enhanced PD-L1 expression (9, 42, 66). While studies highlighting the association between E5 oncoprotein of high-risk HPV and PD-1/PD-L1 expression in cervical cancer are scarce, Kim et al. investigated the effects of E5 expression on epidermal growth factor receptor-1 (EGFR1) and vascular endothelial growth factor (VEGF) in cervical cancer cell lines (12), concluding that E5 oncoprotein activates EGFR1 thereby upregulating the expression of VEGF (9). Furthermore, HPV16-E6 binds to Na^+^/H^+^ exchanger regulatory factor-1 (NHERF-1) and results in the breakdown of NHERF-1 *via* the proteasome pathway (67). Also, E7 combines with E6 to underpin NHERF-1 degradation, thus triggering the PI3K/AKT pathway (68). Moreover, *in vitro* data link loss of NHERF-1 in cervical cancer with increase in cellular growth, proliferation, cell cycle, and ERK signaling stimulated by EGFR (69, 70). Incessant EGFR activation by NHERF-1 correlates with poor prognosis in cervical cancer (70). Also, NHERF-1 has been found to play a role in cisplatin resistance of cervical cancer cells by inhibiting AKT and ERK signaling pathways (71). Moreover, loss of NHERF-1 expression in the cervical cell line promoted cellular adhesion, wound healing, and invasion, indicating NHERF-1 as a plausible therapeutic target for cervical cancer patients (72). Similarly, He et al. report that EGFR activation increases the expression of yes-associated protein (YAP), thus inducing cervical cancer cell proliferation and migration (73). More importantly, Chen et al. investigated the effects of EGFR1 on PD-L1 activity in NSCLC (74). This study found that EGFR1 activation induces PD-L1 expression through the phosphorylation of ERK1/2/c-Jun, potentially causing T cell apoptosis. On the other hand, Lee et al. analyzed the role YAP plays in the activation of the PD-1/PD-L1 pathway (75). The study concluded that YAP regulates the transcription of PD-L1; thus, targeting PD-L1 either directly or through YAP could be a potential therapeutic target (75). Yang et al. showed a correlation between increased PD-1 expression on T cells and persistent high-risk HPV infection with the development of cervical intraepithelial neoplasia (CIN) (44). Altogether, E5 oncoprotein activates EGFR, which enhances YAP leading to the upregulation of PD-L1, thus initiating T cell apoptosis and persistent HPV infections and augmenting the risk of cervical cancer development (44, 73, 75). MicroRNAs (miRs), a class of small regulatory, non-coding RNA molecules, play a vital role in gene expression modulation and regulating major pathways (76). High-risk HPV oncoproteins interact with miRs to regulate the onset and progression of cervical cancer (77). One such miR, miR-34a, is directly regulated by p53 and targets molecules regulating cell proliferation, cellular apoptosis, G1 arrest, DNA repair, and senescence (78–81). HPV-E6 blocks miR-34a expression *via* the p53 pathway inducing virus-infected cell survival and cancer cell proliferation and metastasis (82, 83). A study by Li et al. (81) showed loss of miR-34a expression in precancerous cervical lesions, indicating this as an early-onset event in cervical cancer development (81). Several studies have shown reduced miR-34a expression in both cervical cancer cells and tissues associated with invasive carcinomas (81, 82, 84–86). Moreover, Cortez et al. (87) and Wang et al. (88) analyzed the role of miR-24a in controlling PD-L1 activity (87). *In vitro* and *in vivo* data showed that p53 regulates PD-L1 *via* miR-34a (87, 88). miR-34a directly attaches to the PD-L1 3′-untranslated region, inhibiting PD-L1; thus, induction of miR-34a resulted in reduced PD-L1-specific T cell apoptosis, indicating that tumor immune evasion is regulated by the p53/miR-34a/PDL1 axis (87, 88). Expression of another miR, miR-200c, was lost in cervical cancer tissue samples and cell lines (89). Elevated p53 levels inhibit miR-200c levels and correlate with increased cervical cell invasion, migration, and proliferation abilities (90, 91). Further, miR-200c expression is significantly reduced in PD-L1-positive samples, suggesting miR-200c as a potential regulator of PD-L1 (92). Thus, increased PD-L1 expression can elevate miR-200c expression, inhibiting tumor cell migration, and metastasis (93).

Moreover, a study by Liu et al. investigated the mechanisms underlying HPV-induced evasion of cervical cancer cells from the host immune system via the PD-L1/PD-1 signaling pathway (66). Quantification of HPV16-E7 expression using immunohistochemistry and RT-PCR analyses revealed low levels of HPV16-E7 in normal tissues in comparison to elevated levels detected in cervical cancer tissues; immunohistochemical analysis showed strong PD-L1 expression in cervical cancer samples (*P* = 0.017) (66). A statistically significant (*P* = 0.043) positive correlation between HPV16-E7 and PD-L1 protein expression in cervical cancer tissues was reported, indicating that HPV16-E7 potentially downregulates lymphocyte proliferation by stimulating the PD-L1 pathway and weakening the immune response to tumor cells (66). Also, the association of HPV-E7 to pRb results in the degradation of the cell cycle allowing cells to undergo unchecked proliferation (94, 95). E7 also binds to and inhibits the IκB complex, thus reducing the levels of NFκB (96). HPV16-E6 and E7 proteins repress basal and TNF-α-inducible NF-kB activity in cervical cancer cells, promoting onset of cervical cancer (97). Loss of NF-kB allows the virus to restrict the host immune response and stimulates continuous HPV infection which can in turn trigger clonal growth and immortalization of cervical epithelial cells (97). Furthermore, PD-L1 expression is regulated by NF-κB during EMT signaling; NF-κB activates PD-L1, promoting cancer growth, and progression (98). Herein it is important to highlight that the interaction between PD-L1 and HPV oncoproteins could be specific to only certain types of high-risk HPVs or coinfection with more than one type since more than 96% of human cervical cancers are positive for HPVs, while around 40% of these cancer cases overexpress PD-L1, as reported by Meng et al. (39) and Feng et al. (43).

Furthermore, numerous studies investigated the role of PD-L1 expression in the prognosis and therapeutic efficacy of cervical cancer patients. Yang et al. explored the correlation between the modulation of the PD-1/PD-L1 axis and CIN grading of cervical cells in high-risk HPV (–) and (+) women (44). In patients with high-risk HPV, ELISA revealed a reduced expression of Th1 cytokines, interferon-c (IFN-c), and interleukin-12 (IL-12). In contrast, Th2-type cytokine and interleukin-10 (IL-10) were increased as compared with high-risk (–) HPV. High-risk HPV positivity correlated with increasing CIN grade. As for the PD-1/PD-L1 axis, Yang et al. showed that the activation of the PD-1/PD-L1 pathway suppresses cell-mediated immunity (CMI), by inhibiting pro-inflammatory cytokines and upregulating anti-inflammatory cytokines, thus leading to localized immunosuppression and high-risk HPV-induced CIN progression (44) (Figure 1). A study by Qin et al. (99) showed that HPV-induced somatic mutations played a vital role in the inhibitory tumor microenvironment resulting in aberrant expression of checkpoint-related genes (CTLA-4, PD-1, and PD-L1) (99). Furthermore, the expression of HPV oncoprotein-associated master regulators was determined and their effect on the functionality of the immune system in cervical cancer was analyzed (99); thus, a positive correlation (*p* < 0.001) was reported between certain HPV-induced master regulators, specifically ENO1, FOSB, PA2G4, SOX9, TEAD4, FOXO4, and MNT, in cervical cancer samples (*n* = 306) in comparison with normal cervix (*n* = 11) tissue obtained from The Cancer Genome Atlas (TCGA). This proposes the induction of an immune-deficient state caused by the HPVE6/E7 expression that is mediated through a variety of pathways including the PD-1/PD-L1 axis (99). In summary, a positive correlation between HPV E5, E6, and E7 oncoproteins and enhanced PD-1/PD-L1 expression has been reported with tumor metastasis (22), tumor progression (100), and poor prognosis (47) in cervical cancer. However, the exact underlying mechanism by which these two elements are interconnected still remains nascent. Studies suggest that E6/E7 activates the PD-1/PD-L1 axis causing an increase in Th2-type cytokine and IL-10 expression and a decrease in Th1 cytokine IFN-c and IL-12 expression leading to immunosuppression and further progression of CIN (101, 102) (Figure 1).

To identify therapeutic targets for local immune modulation, multiparameter flow cytometric T-cell profiling of primary cervical tumors (PT) and tumor-draining lymph nodes (TDLN) of cervical cancer was performed (103). The study reported that the inhibition of PD-1 led to a significant increase in E6-specific T-cell responses in 80% of HPV16+ TDLN and in 20% of HPV16+ PT as well as enhanced expression levels of CD8+, FoxP3+, and CD25+ T cells, thus confirming its efficacy (103). Taking into account the promising results reported from PD-1/PD-L1 inhibitors in the treatment of high-risk HPV-mediated cervical cancers (104), the efforts are now directed toward combination therapy with PD-1 pathway blockers and E6/E7-targeted therapy. Effects of CRISPR/Cas9 to target both HPV and PD-1 *in vitro* and *in vivo* models (104) were tested, and results showed that the combination therapy with gRNA-PD-1 and gRNA-HPV16 E6/E7 significantly reduced tumor growth and enhanced survival. Moreover, an increase in the population of dendritic cells, CD8+, and CD4+ T lymphocyte was observed following the administration of the combination therapy (104). Ongoing clinical trials are investigating the use of various PD-1 inhibitors/modulators [nivolumab (105), atezolizumab (106), and pembrolizumab (107)] in combination with concurrent chemoradiation therapy (CCRT) for the management of advanced and recurrent cervical cancers. Additionally, promising results from a phase II clinical trial investigating the effects of combined immune checkpoint inhibitor and tumor-specific vaccine (ISA 101, a synthetic long-peptide HPV-16 vaccine) for patients with incurable HPV-16-induced cancer was obtained (108).

These investigations show that PD-1/PD-L1 inhibitors could be in fact promising therapeutic targets for HPV-associated cancers, especially cervical. Although studies analyzing the potency of PD-1/PD-L1 inhibitors in the management of cervical cancer are rare, there are few studies on the clinical application of PD-1/PD-L1 inhibitors and/or new inhibitors targeting other elements of cell death program. Given the promising results of these preliminary studies, we believe more investigations are necessary to elucidate the role and efficacy of such treatments. The major limitations in studies conducted thus far include lack of data regarding overall survival, drug resistance, and the underlying mechanisms. Such data can aid in selecting patients for cell death program inhibitors, including PD-1/PD-L1, and pave the way for new therapeutic interventions. On the other hand, it is important to highlight that PD-1/PD-L1 inhibitors have proven to be a viable therapy that can reactivate immune response against cancerous cells and favor apoptosis as opposed to tumor growth and proliferation. Moreover, current clinical trials investigating the use of cell death program inhibitors, including PD-1/PD-L1, for the treatment of cervical cancer are limited to advanced, persistent, and metastatic cancers. Therefore, it would be interesting to investigate the effectiveness of these agents on early-stage cervical cancers in a small cohort. Additionally, the promising outcomes noted in combination therapy of PD-1 inhibitors with other treatment modalities in various types of cancers favor combination therapy as opposed to PD-1 inhibitor monotherapy for cervical cancer management.

## Conclusion

It is known that the majority of human cervical cancers are positive for high-risk HPVs that involve three known oncoproteins, E5, E6, and E7, which regulate cell-cycle and tumor-suppressor genes, thereby affecting apoptosis and cell death program. We have reviewed the cross talk between high-risk HPV oncoproteins and the PD-1/PD-L1 pathway in the pathogenesis of cervical cancer. It is evident that there is a strong interplay between oncoproteins of high-risk HPV and cell death, including PD-1 activation, as reported in several investigations. More specifically, HPV oncoproteins E5 and E6/E7 can activate the PD-1/PD-L1 axis; hence, further research is required to elucidate the association between HPV status and the efficacy of PD-1/PD-L1 inhibitors and/or other inhibitors related to cell death program in cervical cancer. On the other hand, analyzing the interaction between oncoproteins of high-risk HPVs and cell death program including PD-1/PD-L1 can also help in identifying candidate novel biomarkers that can help in the prognosis of cervical cancer patients.

## Author Contributions

All authors have read and agreed to the published version of the manuscript.

## Conflict of Interest

The authors declare that the research was conducted in the absence of any commercial or financial relationships that could be construed as a potential conflict of interest.
